# SHAPER: A Web Server for Fast and Accurate SHAPE Reactivity Prediction

**DOI:** 10.3389/fmolb.2021.721955

**Published:** 2021-07-28

**Authors:** Yuanzhe Zhou, Jun Li, Travis Hurst, Shi-Jie Chen

**Affiliations:** ^1^ Department of Physics and Astronomy, University of Missouri, Columbia, MO, United States; ^2^ Department of Biochemistry, University of Missouri, Columbia, MO, United States; ^3^ Institute of Data Sciences and Informatics, University of Missouri, Columbia, MO, United States

**Keywords:** SHAPE reactivity prediction, structure prediction, structure analysis, web server, coarse-grained simulation

## Abstract

Selective 2′-hydroxyl acylation analyzed by primer extension (SHAPE) chemical probing serves as a convenient and efficient experiment technique for providing information about RNA local flexibility. The local structural information contained in SHAPE reactivity data can be used as constraints in 2D/3D structure predictions. Here, we present SHAPE predictoR (SHAPER), a web server for fast and accurate SHAPE reactivity prediction. The main purpose of the SHAPER web server is to provide a portal that uses experimental SHAPE data to refine 2D/3D RNA structure selection. Input structures for the SHAPER server can be obtained through experimental or computational modeling. The SHAPER server can accept RNA structures with single or multiple conformations, and the predicted SHAPE profile and correlation with experimental SHAPE data (if provided) for each conformation can be freely downloaded through the web portal. The SHAPER web server is available at http://rna.physics.missouri.edu/shaper/.

## 1 Introduction

With the development of novel ribonucleic acid (RNA) structure determination methods alongside discoveries of new RNA structures and cellular functions, RNA has become increasingly important, contributing new avenues in the development of therapeutic applications for human diseases. Computational modeling of RNA structures could greatly deepen our understanding of RNA folding mechanisms. However, computational prediction of RNA structures from the sequence remains a significant unsolved problem (Shapiro et al., 2007; Laing and Schlick, 2010; Miao and Westhof, 2017).

Although lacking complete structural information, some experimental methods can provide useful details for guiding structure prediction. The selective 2′-hydroxyl acylation analyzed by primer extension (SHAPE) method is a convenient and efficient RNA structure probing technology with single nucleotide resolution that can provide information about local nucleotide structural dynamics (Merino et al., 2005; Wilkinson et al., 2006). The SHAPE reactivity of a nucleotide is reflected by the ability to bind SHAPE reagents—small ligands such as 1-methyl-7-nitroisatoic anhydride (1M7)—that preferentially bind to the oxygen of 2′-hydroxyl group of RNA nucleotides (Lee et al., 2017). Previous studies (Gherghe et al., 2008; Weeks, 2010; McGinnis et al., 2012) suggested that SHAPE reactivity is correlated with nucleotide flexibility, where unconstrained nucleotides tend to be more reactive while nucleotides constrained by base pairing, stacking, or other interactions are less reactive. The signals seen in SHAPE experiments intrinsically reflect interactions in the 3D structure, and can therefore be used to place effective constraints on the possible structures in a conformational pool generated by computational modeling software.

**FIGURE 1:**
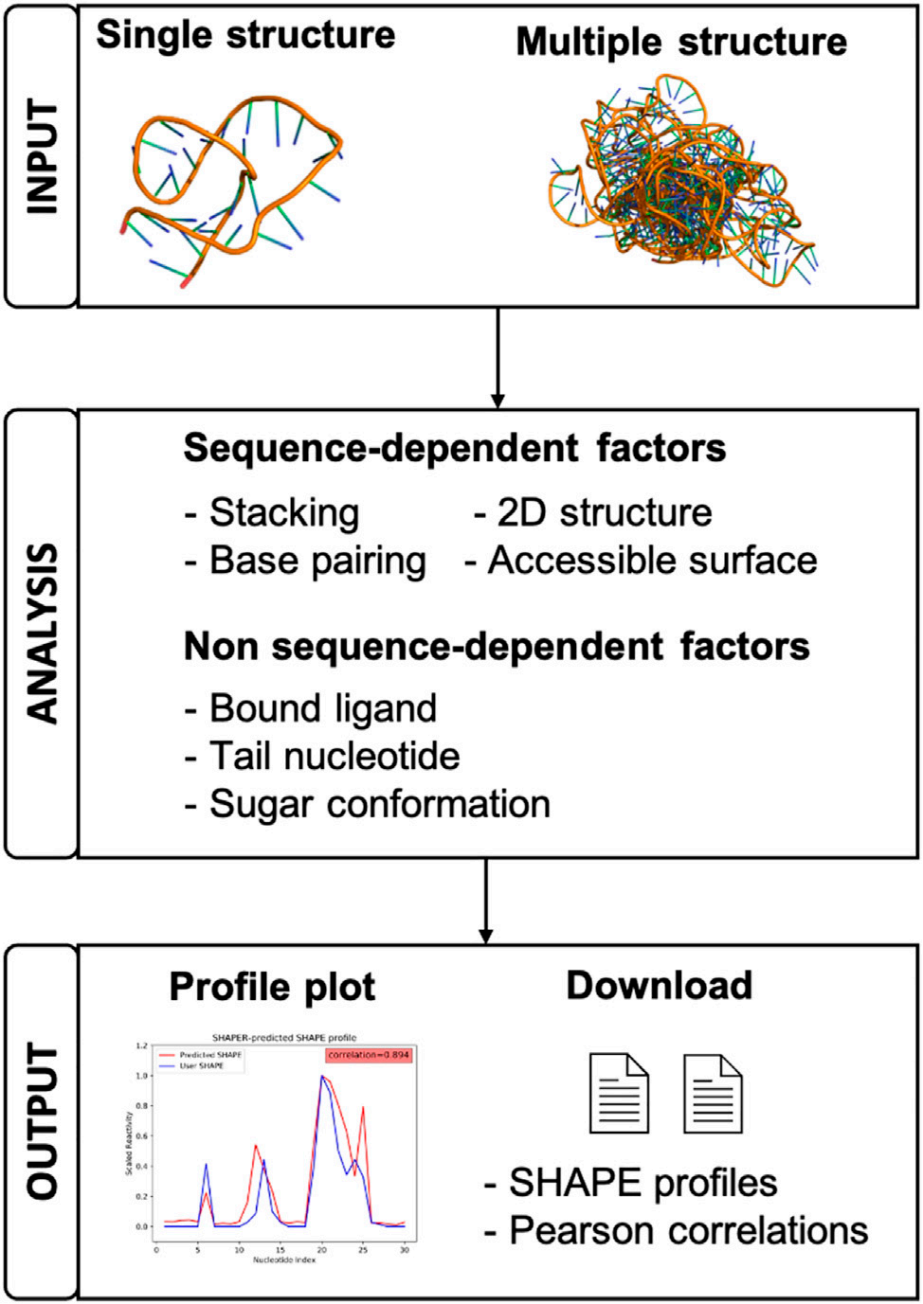
A schematic view of the organization and function of the SHAPER web server.

Since many RNA structure prediction studies would benefit from utilizing experimental SHAPE data, having a freely available, dedicated web server for rapidly predicting SHAPE profiles and filtering structural ensembles is essential. In this paper, we present our SHAPE predictoR (SHAPER) web server for predicting the SHAPE profile of any given RNA structure. The organization of the server is shown in Figure 1. The SHAPER server only requires the 3D coordinates of the target RNA (in PDB format). These structures can come from experimental structures, simulation snapshots, or computational structure-prediction models, etc. The SHAPER server can accept either individual structures or a structural ensemble, and the output contains predicted SHAPE profiles with the correlations between predicted profiles and a provided experimental SHAPE profile (if available). The engine powering the SHAPER web server is the new SHAPE prediction model (Hurst and Chen, 2021), which is an updated version of the original 3D Structure-SHAPE Relationship (3DSSR) model (Hurst et al., 2018). The SHAPER model incorporates RNA sequence-dependent bias into the prediction and is able to provide higher correlations between SHAPE data and the native RNA structure, which improves our ability to discern between SHAPE-compatible and -incompatible structures on decoys than the previous 3DSSR model.

## 2 Materials and Methods

### 2.1 Workflow

The following shows both the workflow and theoretical background of the SHAPER server. Detailed description and analysis of the SHAPER model can be found in the original paper (Hurst and Chen, 2021).

#### 2.1.1 Step 1: Uploading Input Data

**FIGURE 2:**
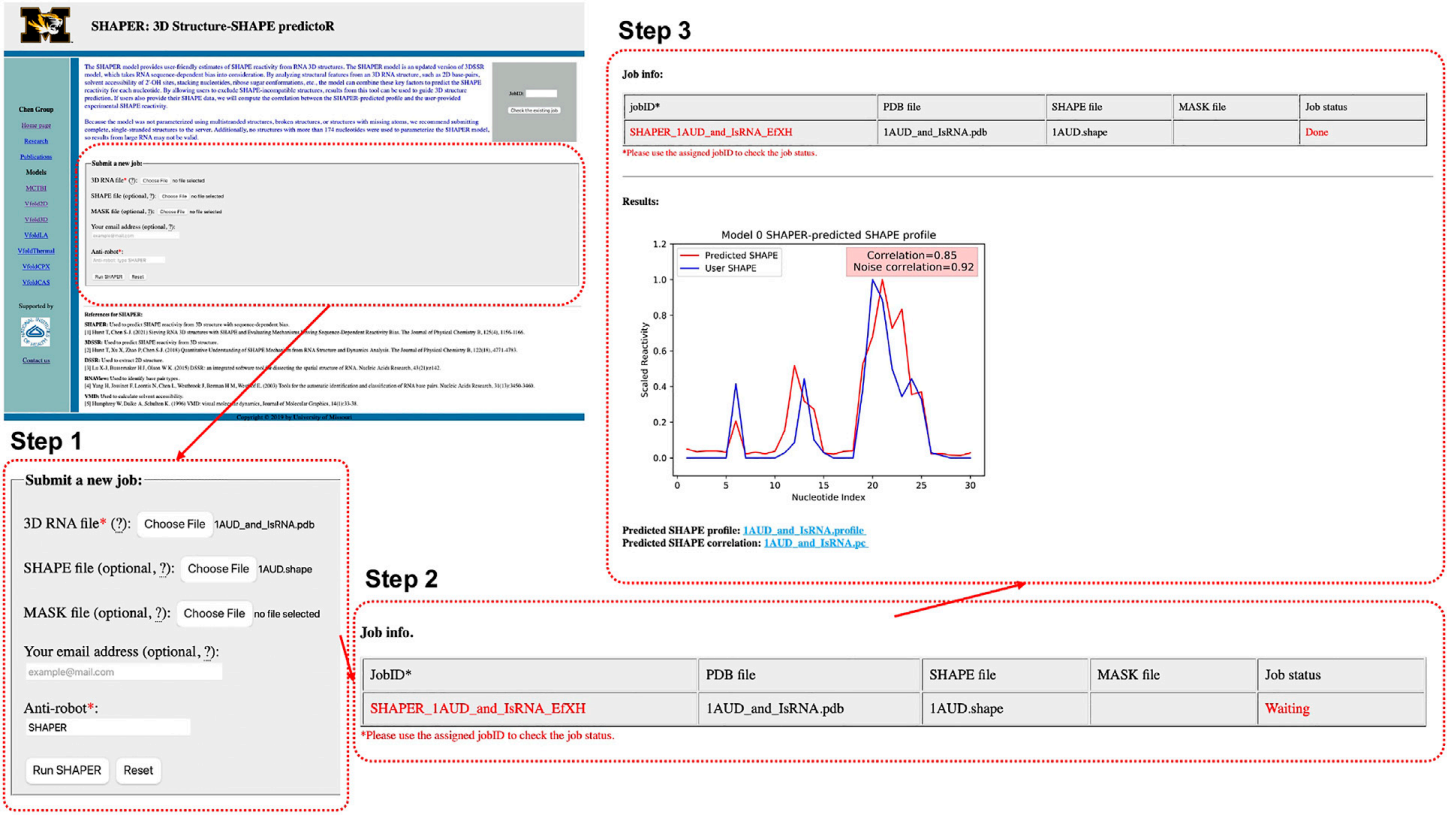
Interface of the SHAPER web server and the steps involved in submitting a job. The overview of the interface of the starting page is shown in the top left, and the area within the dashed red box is updated in each step. There are three steps: uploading and choosing parameters (Step 1), waiting the job (Step 2), and checking results of the job (Step 3).

As shown in Figure 2, Step 1, the input parameters are the following: (1) the input RNA structure file in PDB format, (2) user provided SHAPE profile, (3) user provided MASK file for the target RNA (for masking nucleotides that interact with ligands), (4) an email address for delivery of the calculation results, and (5) a simple text verification to prevent robotic usage. Required parameters are labeled by red asterisks. After submitting the job, the user will be redirected to a waiting page (Figure 2, Step 2), where they can view information about the running job. The information shown in the table in Figure 2, Step 2 includes: JobID—an identification code used to look up the results—and the file names of the RNA, SHAPE, and MASK file uploaded by user, respectively.

#### 2.1.2 Step 2: Calculating SHAPE on Server Side

After submitting the job, SHAPER will put the job in a queue and will run the job once the computational resources are available. Usually, it takes less than a minute for a single structure with around 100 nucleotides. The procedures taken by the SHAPER server are listed in the order of execution.

• *Validating input*.

The input RNA file (in PDB format) is checked before any further processing. Entities other than RNA will be removed from the PDB file, only the backbone of modified residues and the first occurrence of atoms with multiple alternative locations will be kept for SHAPE reactivity calculations.

• *Identifying base pairing and stacking interactions*.

Base pairs are identified by RNAView (Yang et al., 2003), while stacking nucleotides are identified by our in-house Perl script. Then, pairing and stacking energies are combined into the interaction energy score (IE) for a given nucleotide *i* as

$$E_{\text{IE}}\left(i\right)=\underset{m}{\sum }\left[A\cdot E_{\text{bp}}^{\left(t\right)}\left(i,m\right)+B\right]+\underset{k}{\sum }E_{\text{st}}^{\left(i\right)}\left(i,k\right)$$ (1)

where all the type-*t* base pairing energies $E_{bp}^{\left(t\right)}\left(i,m\right)$ and all the stacking energies $E_{st}^{\left(i\right)}\left(i,k\right)$ of nucleotide *i* are summed together. *A* and *B* are two extra parameters trained for the SHAPER model. The base pairing interactions $E_{bp}^{\left(t\right)}\left(i,m\right)$ were derived through a quasi-chemical statistical potential approach based on the statistical frequencies of the base pairing interactions extracted from the non-redundant RNA Basepair Catalog (Narayanan et al., 2013), and the stacking energies introduce 5′ → 3′ polarity-dependence by using different weights and energy parameters for upstream 5′ and downstream 3′ nucleotides, respectively.

• *Extracting 2D structure*.

Using the Dissecting the Spatial Structure of RNA (DSSR) tool (Lu et al., 2015), the 2D structure is extracted from the input 3D structure. A parameter *E*_2*D*_(*i*) is introduced to represent the energy contributed by the base pairing nucleotide *i* in the 2D structure.

• *Accounting for other structural features.*

(1) Ligand Accessible Surface (*A*_*SAS*_). The accessibility of the SHAPE reagent (1M7) to the 2′-hydroxyl of each nucleotide is calculated using Visual Molecular Dynamics (VMD) (Humphrey et al., 1996) with a bead radius of 2.0 Å.

(2) Ribose sugar conformations. Previous studies (Vicens et al., 2007; Frezza et al., 2019) suggest that the conformation of the ribose sugar is important for SHAPE-reactivity. A correction *F*_*sug*_ determined by the pseudorotation angle of the ribose is employed to account for this effect.

(3) Tail nucleotides. Simple parameter *F*_*term*_ on terminal nucleotide is used to account for the effect of the short nucleotide sequence added at the terminal regions during SHAPE experiments.

(4) Bound ligands. Nucleotides interacting with a bound ligand need different treatment. To account for these effects, a ligand binding energy penalty *E*_*lig*_ is introduced for the nucleotides that are interacting with bound ligands. This is achieved by masking the nucleotides that interact with the ligand. Users can supply their own mask file when submitting jobs on the web server, supplying 0 and 1 for non-interacting and interacting nucleotides, respectively. By default, the SHAPER server will treat all nucleotides as not interacting with ligand.

• *Accounting for the effects of neighboring nucleotides.*

Due to observations that a free nucleotide next to rigid nucleotides will be less reactive than a free nucleotide that has flexible neighbors, we introduce a weighted averaging scheme to account for this type of correlative effect for *E*_*IE*_, *E*_2*D*_, and *A*_*SAS*_ terms as

$${\overset{‐}{E}}_{\text{IE}}\left(i\right)=\frac{\overset{3}{\underset{j=0}{\sum }}w_{j}\times E_{\text{IE}}\left(i+j-1\right)}{\overset{3}{\underset{j=0}{\sum }}w_{j}}$$ (2)

$${\overset{‐}{E}}_{2\text{D}}\left(i\right)=\frac{\overset{3}{\underset{j=0}{\sum }}d_{j}\times E_{2D}\left(i+j-1\right)}{\overset{3}{\underset{j=0}{\sum }}d_{j}}$$ (3)

$${\overset{‐}{A}}_{\text{SAS}}\left(i\right)=\frac{\overset{3}{\underset{j=0}{\sum }}a_{j}\times A_{\text{SAS}}\left(i+j-1\right)}{\overset{3}{\underset{j=0}{\sum }}a_{j}}$$ (4)

where *w*_0_ − *w*_3_, *d*_0_ − *d*_3_, and *a*_0_ − *a*_3_ are weights accounting for the influence of interactions involving the nucleotide of interest (NOI) and/or neighboring nucleotides.

• *Predicting the SHAPE profile*.

The final SHAPE prediction is a combination of the interaction factors, written as

$$p_{i}=SF_{i}\times e^{SE_{i}}$$ (5)

where structural factors *SF*_*i*_ and energy-like scores *SE*_*i*_ are determined by

$$SF_{i}=\left({\overset{‐}{A}}_{SAS}\left(i\right)+A_{SAS}^{0}\right)\times F_{sug}\left(i\right)\times F_{term}\left(i\right)$$ (6)

$$SE_{i}={\overset{‐}{E}}_{2D}\left(i\right)+{\overset{‐}{E}}_{IE}\left(i\right)+E_{lig}\left(i\right)$$ (7)

and $A_{SAS}^{0}$ is a parameter that accounts for the breathing of the RNA structure that may allow an apparently inaccessible nucleotide to become accessible to the SHAPE reagent. The model implies an effective ambient temperature when modeling SHAPE reactivity. Indeed, solution conditions including temperature can influence RNA conformational fluctuation and the reaction for SHAPE reagents (such as 1-methyl-7-nitroisatoic anhydride) to form 2′-O-adducts with RNA nucleotides. Because SHAPE experimental data were collected under the folding conditions for the respective (folded) RNAs, the parameters in the model may be appropriate for the selection of folded RNA structures for the experimental conditions involved in the training data set. Considering that different SHAPE experimental data for different RNAs were often collected at different solution (such as temperature) conditions, the parameters in the model reflect an average effect of the different experimental conditions.

• *Calculating regular and noise-adjusted Pearson correlations*.

In the original 3DSSR model, the relationship between the predicted SHAPE profile and experimental SHAPE data (if provided) was characterized by the Pearson correlation (PC). However, this regular PC does not account for the log-normality of SHAPE data and noise found by multiple previous studies (Deng et al., 2016; Vaziri et al., 2018). The newer SHAPER model uses a noise-adjusted normalization method to calculate the noise-adjusted PC between the predicted SHAPE profile and reweighted experimental SHAPE reactivities (Hurst and Chen, 2021).

#### 2.1.3 Step 3: Showing Output

After submitting a job, the user will be directed to a result page which shows the job status and information about the input files. This page will be refreshed every few seconds. Once the job is done, The result page will be updated and the Job status will change from “Waiting” to “Done”. A plot of the predicted and user-provided (if any) SHAPE profile along with normal and noise-adjusted Pearson correlation coefficients will appear below the status table. Links to download corresponding SHAPE prediction and correlation files will appear at the bottom of the page (Figure 2 Step 3). Existing results can be accessed by using the JobID, by bookmarking the address of the result page, or by checking email results (if provided).

### 2.2 Server Implementation

Several programming and scripting languages are used in the SHAPER server, including Bash, C++, Python, Perl, and Tcl. The SHAPE prediction module is implemented in C++ for performance. Third party software packages are used in other modules for preparing the necessary input files. Dissecting the Spatial Structure of RNA (DSSR) (Lu et al., 2015) is used to extract the 2D structure and torsional information of the ribose sugars from a 3D structure. RNAView (Yang et al., 2003) is used to identify base pair types shown in 3D structure, and the identification of stacking interactions is carried out by our in-house program written in Perl. The ligand accessible surface of the 2′-hydroxyl for each nucleotide is calculated using Visual Molecular Dynamics (VMD) (Humphrey et al., 1996). The above tools help automate the preparation process and greatly reduce the potential for human error. All modules were combined by Python and the web server is based on Apache 2.2.15.

## 3 Case Study

### 3.1 Sieving RNA 3D Structures Generated by 3D Structure Prediction Software

**FIGURE 3:**
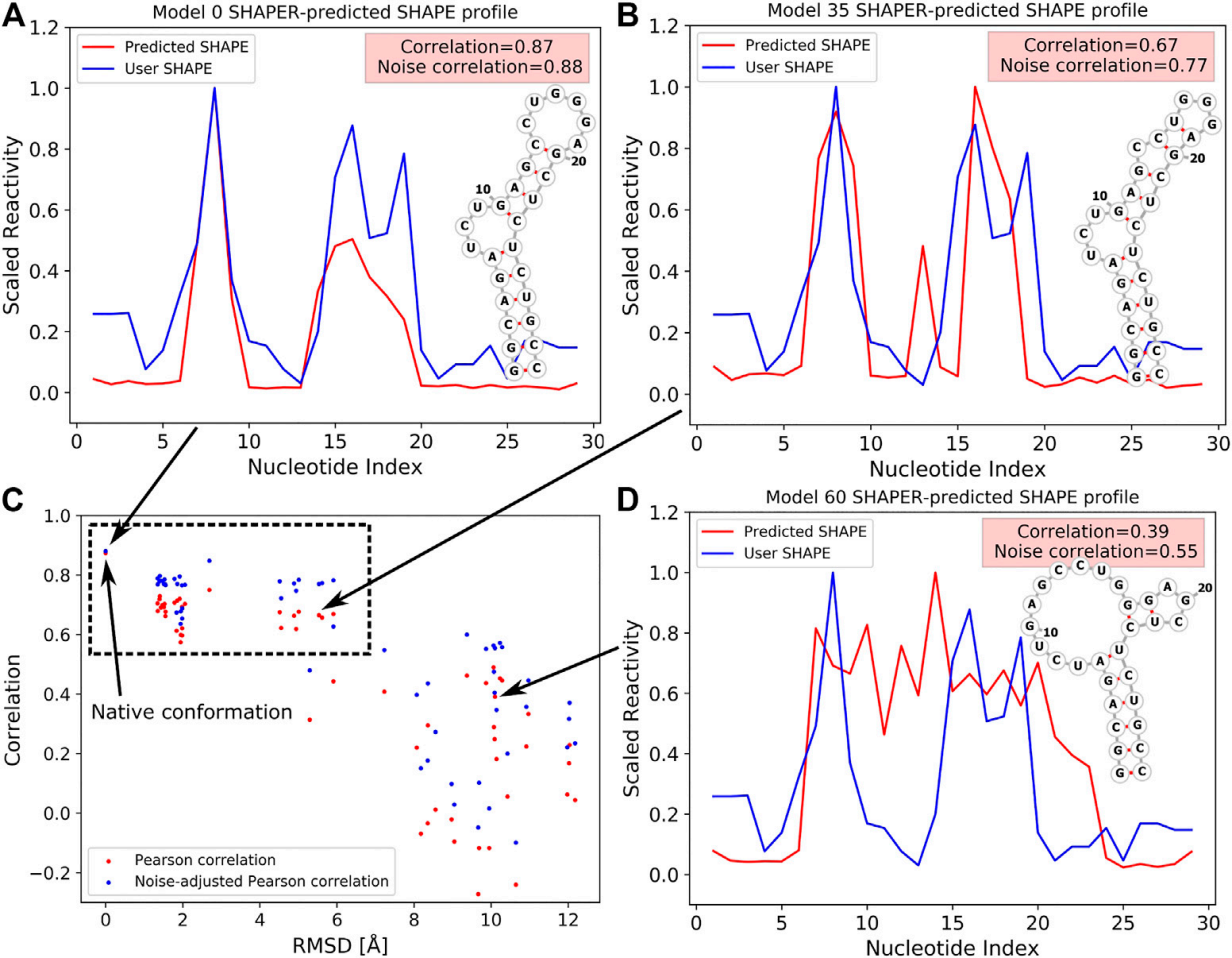
SHAPE profiles for native **(A)** and selected decoy **(B,D)** conformations at different RMSDs. Predicted and experimental (i.e., User SHAPE) profiles are shown in red and blue curves, respectively. 2D structure in **(A)** corresponds to the native structure, and 2D structures shown in **(B,D)** were used as constraints to run the simulations. **(C)** The relation between PC/noise-adjusted PC (red/blue) and RMSDs relative to the native structure for 60 tested conformations (include the native structure, PDB code: 2L8H). The data point of the native conformation is shown on the top left of **(C)** and pointed out by an arrow.

To better illustrate the function of the SHAPER web server, we ran an example case with known experimental SHAPE data to show the ability of SHAPER to distinguish near-native conformations from a pool of decoys. The test RNA structure (PDB code: 2L8H) contains 29 nucleotides. We used our coarse grained (CG) simulation software (IsRNA) (Zhang and Chen, 2018; Zhang et al., 2021) and an all-atom molecular dynamics (MD) simulation to generate 59 decoy conformations for the target RNA. We selected 20 near-native conformations and 39 non-native conformations generated with native and non-native 2D structures (Hurst and Chen, 2021). These decoys along with the native structure allow us to show the ability of the SHAPER server to distinguish native conformation from conformational pools. Then we put these 60 structures into the SHAPER web server, and the SHAPE correlation coefficients (PC and noise-adjusted PC) between predicted SHAPE profiles and experimental SHAPE data were calculated. The root mean square deviations (RMSDs) between the native and decoy conformations were calculated for heavy atoms. As shown in Figure 3C for the relationship between RMSD and SHAPE correlation coefficients, the native structure shows the highest correlation, and the near-native conformations around 2 Å of RMSD also have high correlations. However, similar correlations were also found for non-native conformations around 4 to 6 Å. This is because the 2D structural constraints (see, Figure 3B) used to generate these decoys are similar to the native 2D structural constraints (see, Figure 3A). As for the non-native conformations generated by using different 2D structural constraints (see, Figure 3B), both correlation coefficients (PC and noise-adjusted PC) drop significantly relative to the values of the native conformation. The above results suggest that SHAPE correlation may serve as a useful measure to sieve structures and find the native and near-native 2D and 3D structures.

## 4 Conclusion

SHAPER is a fast and accurate web server to predict SHAPE profile for any given RNA structure. Compared to the original 3DSSR model, SHAPER greatly improves performance (Hurst et al., 2018) by accounting for sequence-dependent bias, tail effects, and ligand binding. In addition, the SHAPER model better reflects that SHAPE reactivities are a direct reflection of the underlying system energetics and incorporates effects related to the log-normality of SHAPE data and noise. The server provides functionalities for predicting SHAPE profiles for RNA with either a single structure or a structural ensemble. Combined with the available experimental SHAPE data, SHAPER can provide a reliable measure of the nativeness of the target conformation and serves as a convenient tool to help researchers select the most probable RNA 3D structures from a pool of decoys.

## Data Availability

The original contributions presented in the study are included in the article/supplementary material, further inquiries can be directed to the corresponding author.
